# Antibodies in the Pathogenesis of Hypertension

**DOI:** 10.1155/2014/504045

**Published:** 2014-06-23

**Authors:** Christopher T. Chan, Maggie Lieu, Ban-Hock Toh, Tin S. Kyaw, Alexander Bobik, Christopher G. Sobey, Grant R. Drummond

**Affiliations:** ^1^Vascular Biology & Immunopharmacology Group, Department of Pharmacology, Monash University, Clayton, VIC 3800, Australia; ^2^Centre for Inflammatory Diseases, Department of Medicine, Southern Clinical School, Monash University, Clayton, VIC, Australia; ^3^Vascular Biology & Atherosclerosis Laboratory, Baker IDI Heart and Diabetes Institute, Melbourne, VIC, Australia

## Abstract

It has long been known that circulating levels of IgG and IgM antibodies are elevated in patients with essential and pregnancy-related hypertension. Recent studies indicate these antibodies target, and in many cases activate, G-protein coupled receptors and ion channels. Prominent among these protein targets are AT_1_ receptors, *α*_1_-adrenoceptors, *β*_1_-adrenoceptors, and L-type voltage operated Ca^2+^ channels, all of which are known to play key roles in the regulation of blood pressure through modulation of vascular tone, cardiac output, and/or Na^+^/water reabsorption in the kidneys. This suggests that elevated antibody production may be a causal mechanism in at least some cases of hypertension. In this brief review, we will further describe the protein targets of the antibodies that are elevated in individuals with essential and pregnancy-related hypertension and the likely pathophysiological consequences of antibody binding to these targets. We will speculate on the potential mechanisms that underlie elevated antibody levels in hypertensive individuals and, finally, we will outline the therapeutic opportunities that could arise with a better understanding of how and why antibodies are produced in hypertension.

## 1. Introduction

Hypertension is defined as chronically elevated blood pressures of >140/90 mmHg. Two of the most common forms of the condition are essential hypertension, where the underlying cause is unknown, and preeclampsia or pregnancy-related hypertension. For several decades it has been known that both essential and pregnancy-related hypertension are associated with elevated serum levels of antibodies [1–4]. More recently, studies in humans and animal models of each condition have begun to identify the protein targets of these antibodies as receptors and ion channels with key roles in the regulation of blood pressure. Such studies not only offer insights into the mechanisms by which antibodies might contribute to hypertension but they also highlight potential new avenues for the clinical management of hypertension. In this brief review we will summarise the evidence in support of a role for antibodies in the pathophysiology of essential hypertension and preeclampsia. We will discuss the protein targets of the antibodies that have been identified in hypertensive individuals and provide some potential explanations for why the production of these antibodies may be elevated. Finally, we will speculate on how such findings may translate into improved clinical management of hypertension.

## 2. Antibodies as Causes of Disease

**Figure 1:**
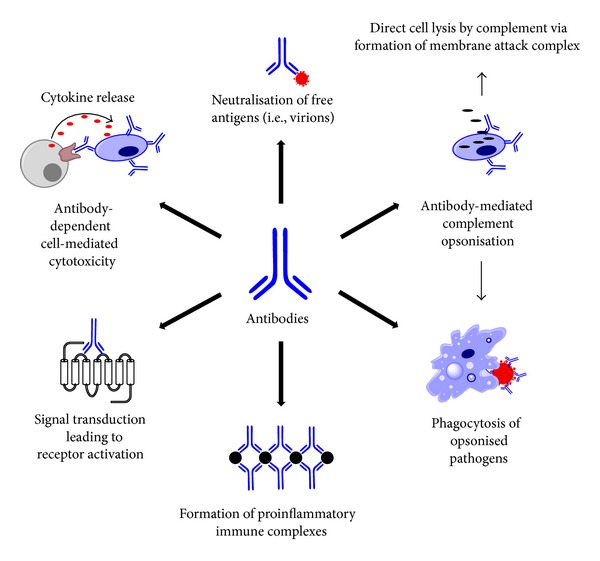
Schematic diagram showing the various types of antibody-mediated autoimmune responses.

Antibodies, or immunoglobulins (Ig), are produced exclusively by B cells as part of the mammalian adaptive immune response [5]. Antibodies play a crucial role in adaptive immunity through their ability to bind antigens, which are normally toxic substances or fragments of pathogen-derived proteins. Such binding results in either neutralisation of the antigen itself or, when the antigen is bound to a cell (e.g., bacteria), destruction of that cell via activation of the complement system, neutrophil degranulation, or phagocytosis by macrophages (Figure 1).

For B cells to generate and secrete antibodies, they must first undergo differentiation into plasma cells. Naïve B cells detect antigens via their B cell receptors, which are membrane-bound immunoglobulins (IgM) with unique and randomly-generated antigen-binding sites [6]. Following binding, the antigen is internalised, processed, and displayed by major histocompatibility class II (MHC II) proteins on the extracellular surface of the B cell [6]. The next step in B cell differentiation involves the detection of the MHC II-presented antigen by the T cell receptor (TCR) of an activated T helper (T_H_) cell that has previously encountered the same antigen. The T_H_ cell also provides essential costimulatory signals in the form of interactions between its CD40 ligand and the CD40 receptor on the B cell [7], as well as cytokines such as interferon-gamma and interleukin-4 [8]. Together, these signals ultimately promote the differentiation of the B cell into an antibody-secreting plasma cell. Such signals also play a crucial role in isotype switching (e.g. IgM → IgG) [9], which allows the generated antibodies to interact with different effector molecules and thereby direct the type of immune response that is mounted.

Although antibodies normally target foreign molecules, under some circumstances they may be raised against host-derived molecules. Such a loss of recognition of “self” is the basis for autoimmune diseases. There are several mechanisms by which aberrant antibody production can lead to autoimmune pathologies (Figure 1). For example, the binding of antibodies to antigens expressed on the surface of endogenous cells may lead to the destruction of these cells via complement- or leukocyte-dependent interactions. This type of response is termed a “Type II Hypersensitivity reaction” and is the cause of the loss of erythrocytes in autoimmune haemolytic anaemia [10]. Alternatively, “Type III Hypersensitivity reactions” involve the recognition of soluble antigens in the host by antibodies and the subsequent formation of “immune complexes.” Immune complexes are cross-linked aggregations of antibodies and antigens that can be deposited in various tissues to cause local inflammatory responses [11]. Immune complexes are a hallmark of several autoimmune disorders including vasculitis and systemic lupus erythematosus where deposition of such complexes in the kidneys gives rise to glomerulonephritis [12, 13]. Finally, some autoimmune diseases are associated with the formation of nonimmunogenic, agonistic antibodies to receptors [14]. These types of diseases are often classified as “Type V Hypersensitivity Reactions.” Agonistic antibodies stimulate receptors in a similar fashion to their cognate ligands and thus lead to overstimulation of the specific system involved. Myasthenia gravis is an example of an autoimmune disease caused by the generation of agonistic antibodies against nicotinic receptors [14, 15].

## 3. Protein Targets of Hypertension-Related Antibodies

Studies dating back to the 1970s demonstrated that essential hypertension in humans is associated with elevated IgG and IgM titres [1–4]. However, these early studies did not identify the targets of these antibodies and thus provided no indication of whether they were important to the pathophysiology of hypertension. Studies from around the same time on animal models confirmed that hypertension was associated with an increase in antibody production and even went some way towards implicating a possible causative role for these antibodies. For example, Ba et al. identified autoantibodies in the serum of spontaneously hypertensive rats (SHRs) that were cytotoxic to T cells. Although the authors did not establish a precise mechanism by which these antibodies contribute to hypertension, they implied that the antibodies might induce apoptosis of “suppressor” T cells that normally prevent damage to the vascular wall and thus protect against cardiovascular disease [16]. In a separate study, it was shown that rats with hypertension induced by renal infarction had high serum levels of antibodies that bound to arteries, glomeruli, and basement membranes of the kidneys [17]. Given the key roles of the kidney and vasculature in the regulation of hemodynamic parameters, this latter study provided an indication that elevated antibody production may actually contribute to the chronic elevation in blood pressure that defines hypertension. And indeed, more recent work identifying the specific molecular targets of the antibodies present in individuals with essential hypertension and preeclampsia not only supports this idea, but also begins to shed light on how elevated antibody production might contribute to elevated blood pressure.

### 3.1. Angiotensin II Type-1 Receptors

The angiotensin II type-1 receptor (AT_1_R) plays a crucial role in the regulation of blood pressure [18]. Stimulation of the AT_1_R by its cognate ligand, angiotensin II, results in vascular smooth muscle cell (VSMC) contraction and proliferation, release of aldosterone from the adrenal glands, and activation of the sympathetic nervous system [18, 19]. Furthermore, it has recently been discovered that AT_1_ receptor activation on T lymphocytes promotes a proinflammatory phenotype that contributes to hypertension [20].

AT_1_R-activating IgG autoantibodies (AT_1_-AAs) directed against the second extracellular loop of the AT_1_R are prevalent in over 95% of patients with pregnancy-associated hypertension, and antibody titres correlate positively with disease severity [21, 22]. AT_1_-AAs appear to activate a cascade of proinflammatory cytokines that contribute directly to hypertension in preeclampsia [23].* In vivo* administration of AT_1_-AAs isolated from preeclamptic humans to pregnant mice was shown to induce hypertension in those animals [24]. Furthermore, AT_1_-AA treatment causes an increase in the circulating levels of tumour necrosis factor-*α* and interleukin-6 in pregnant mice and inhibition of these cytokines with neutralising antibodies blunts hypertension [24, 25].

AT_1_-AAs have also been identified in a subset of individuals with essential hypertension [26–28]. These AT_1_-AAs appear to be similar in function and specificity as those identified in preeclamptic patients as they also bind to the second extracellular loop of the AT_1_R [26, 28]. The fact that essential hypertensive patients with AT_1_-AAs respond with greater blood pressure reductions to AT_1_R blockade by candesartan than hypertensive individuals without AT_1_-AA [29, 30] suggests a causal role for AT_1_-AAs in at least some cases of hypertension.

### 3.2. Alpha-1 Adrenergic Receptors

The alpha-1 adrenergic receptor (*α*_1_AR) is a G-protein coupled receptor that is primarily expressed on VSMCs and proximal renal tubules [31]. Activation of the *α*_1_AR by its endogenous ligands, noradrenaline and adrenaline, or synthetic compounds such as phenylephrine, results in VSMC contraction and increased total peripheral resistance, as well as increased Na^+^ reabsorption in the kidney causing elevated blood pressure [32]. IgG receptor-activating autoantibodies against the *α*_1_AR (*α*_1_AR-AA) have been described in patients with essential hypertension [28, 33–35]. Unlike AT_1_-AAs, which appear to bind to a similar domain of the AT_1_R (i.e., the second extracellular loop) irrespective of the patient from which they were derived, *α*_1_AR-AA from different patients display selectivity towards separate regions of the receptor, with antibodies from some individuals targeting the first extracellular loop, and antibodies from other individuals targeting the second extracellular loop [33, 34]. It is unclear whether these varying binding properties have implications for the ability of a specific antibody to modulate receptor function. It is also unclear whether *α*_1_AR-AAs are present or elevated in individuals with preeclampsia.

### 3.3. Beta-1 Adrenergic Receptors

The *β*-1 adrenergic receptor (*β*_1_AR) shares the same endogenous agonists with *α*_1_ARs but differs in ligand affinity, tissue distribution, and functional outcomes following stimulation. *β*_1_ARs are localised predominately in cardiac tissue where activation results in increased heart rate and contractility and an overall increase in cardiac output [36]. Cardiac output is a major determinant of blood pressure and thus *β*_1_AR blockers have long been used as antihypertensive agents [37]. *β*_1_AR agonistic IgG autoantibodies (*β*_1_AR-AA) against the second extracellular loop of the receptors were detected in the serum of spontaneously hypertensive rats [38]. Furthermore, injection of *β*_1_AR-AAs into healthy Lewis rats promoted cardiomyopathy and increases in systolic blood pressure [39]. Although evidence for the presence of *β*_1_AR-AAs in human essential hypertension and preeclampsia is lacking, these antibodies have been identified in patients with idiopathic dilated cardiomyopathy [40].

### 3.4. L-Type Voltage Gated Calcium Channels

L-type voltage gated calcium channels (L-type VOCCs) are expressed on VSMCs in resistance vessels and, in their open state, directly contribute to vascular tone and blood pressure by facilitating the influx of extracellular Ca^2+^ [41]. It is well established that L-type VOCC expression in the vasculature is upregulated in experimental hypertension and that this contributes to increased Ca^2+^ levels in VSMCs and thus elevated vascular resistance [42–47]. The importance of L-type VOCCs in human hypertension is highlighted by the fact that inhibitors of these channels (e.g., nifedipine) continue to represent one of the most effective and widely-used classes of antihypertensive medications [48]. Thus, it is noteworthy that autoantibodies against L-type VOCCs were identified in some patients with essential hypertension [49]. Although the authors did not examine the effect of these antibodies on L-type VOCC function, a separate study demonstrated increased intracellular Ca^2+^ influx in pancreatic islet cells following the binding of analogous IgG and IgA autoantibodies to L-type VOCCs in the setting of Type-1 diabetes [50]. This implies that antibodies against L-type VOCCs are likely to be agonistic in nature and could thus contribute to increased VSMC Ca^2+^ influx in hypertension.

### 3.5. Heat Shock Proteins

Heat shock proteins (HSPs) are a family of highly-conserved proteins that provide protection against danger-related signals by acting as molecular chaperones to assist in the folding and trafficking of proteins during cellular stress [51]. Among the multitude of known mammalian HSPs, HSP-70 has received most attention in the field of hypertension research. First,* in vitro* exposure of cultured VSMCs, endothelial cells or isolated aortic rings to hypertension-relevant stimuli such as oxidative stress, cyclic strain, and angiotensin II, induces the expression of HSP-70 [52–54]. Second, levels of HSP-70 and HSP-70-reactive CD4 T cells are elevated in the kidneys in several rat models of hypertension [55–57]. Finally, HSP-70 serum concentrations are elevated in pregnancy-associated hypertension and are positively correlated with blood pressure in affected women [58].

Elevated levels of IgG and IgA antibody titres against HSP-70 have been identified in essential hypertensive individuals [59, 60]. Surprisingly, elevated anti-HSP-70 antibody levels in essential hypertension were not associated with changes in serum HSP-70 in these patients [60]. Thus, the function of anti-HSP-70 antibodies in essential hypertension remains unclear. It is possible that anti-HSP-70 antibodies could either promote inflammation via formation of circulating immune complexes or, alternatively, alleviate the proinflammatory actions of these proteins via neutralisation. A more recent study by Molvarec et al. was unable to demonstrate any changes in circulating levels of anti-HSP-70 antibodies in women with preeclampsia [61].

### 3.6. Miscellaneous

A study in borderline hypertensive patients described a reduction in circulating levels of anti-oxidised LDL IgG antibodies [62]. However, a follow-up investigation failed to detect any difference in levels of these antibodies between patients with clinical hypertension and normotensive controls [63], and thus the significance of anti-oxidised LDL antibodies in the pathophysiology of hypertension is unclear. Other studies in borderline hypertensive individuals detected elevations in circulating anti-endothelial cell IgG and IgM antibodies [64–66]. While data in the setting of human essential hypertension is still missing, these antibodies have been identified in women with severe preeclampsia and have been proposed to contribute to endothelial dysfunction [67].

**Figure 2:**
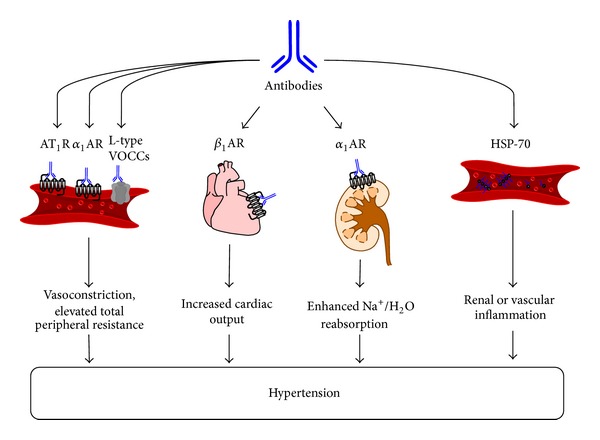
Schematic diagram showing the mechanism by which autoantibodies may promote increases in vascular tone, cardiac output, Na^+^/water reabsorption, and renal and vascular inflammation, and thereby contribute to hypertension.

Figure 2 provides a summary of the targets of antibodies that have been shown to be elevated in hypertension and the potential mechanisms by which these antibodies may contribute to disease pathophysiology.

## 4. Mechanisms Contributing to Antibody Production

The previous discussion highlighting the association of hypertension with increased antibody levels raises the question: what are the mechanisms involved in antibody production during hypertension? There are at least three possible explanations including (1) neoantigen formation; (2) molecular mimicry; and/or (3) aberrant B cell function.

### 4.1. Neoantigen Formation

Harrison and colleagues recently put forward a hypothesis whereby “neoantigens” were highlighted as the central mediators of the immune cell activation that underlies hypertension [68, 69]. These authors suggested that hypertensive stimuli such as Ang II, catecholamines, and aldosterone initially induce a moderate increase in blood pressure via their “classical” actions in promoting Na^+^/water retention, vasoconstriction, and/or increased sympathetic drive [68, 69]. This moderate increase in blood pressure is postulated to cause both mechanical and oxidative stress in the walls of blood vessels and also in the kidneys, leading to structural and chemical modifications to proteins such that they are no longer recognised as “self,” but rather as neoantigens. These neoantigens are predicted to then invoke an adaptive immune response, leading to vascular and renal inflammation and exacerbation of hypertension [68, 69]. However, it is presently unclear whether any of the proteins that have been identified as targets of antibodies in hypertensive animals and humans (e.g., AT_1_R, *α*_1_AR, *β*_1_AR, L-type VOCCs, or HSP-70) undergo structural or chemical alterations that may render them as potential neoantigens.

### 4.2. Molecular Mimicry

Another possible explanation for autoantibody production in hypertension involves molecular mimicry, where foreign or pathogen-derived antigens trigger an immune response against “self” peptides of similar homology [70]. A prominent example of this is myasthenia gravis, an autoimmune disease where agonistic antibodies are raised against nicotinic receptors [14, 15]. These antibodies show strong cross-reactivity to herpes simplex virus glycoprotein D [15].

Relating to hypertension, AT_1_-AAs from women with preeclampsia were shown to recognise the VP2 caspid protein from parvovirus B19 [71]. The seroprevalence of this virus has been reported to be more than 70% of the adult population [72, 73], and its involvement in predisposing infected individuals to various autoimmune disorders has been recognised [73]. Thus, it is plausible that molecular mimicry underlies the elevations in AT_1_-AAs observed in preeclampsia [74] and essential hypertension.

Although not examined in the setting of hypertension, there is evidence in other disease states that antibodies against L-type VOCCs and *β*_1_ARs may also arise as a result of molecular mimicry. For example, autoantibodies against L-type VOCCs that are present in a subset of individuals with Type-1 diabetes also recognise the B4 VP1 protein of the coxsackievirus [50]. Interestingly, the seroprevalence of coxsackievirus infection was reported to positively associate with the incidence of hypertension in a Chinese Mongolian population [75]. Furthermore, *β*_1_AR-AAs were demonstrated to recognise the carboxy-terminus of the ribosomal P0 and P2 proteins from* Trypanosoma cruzi, *the parasite that is responsible for chronic Chagas heart disease [76–79]. Conversely, autoantibodies against human ribosomal P proteins that are present in patients with systemic lupus erythematosus cross-react with (but do not activate) the *β*_1_AR [78]. These findings may suggest that a high degree of sequence and/or structural homology exists between *β*_1_AR and ribosomal P proteins.

### 4.3. Aberrant B Cell Function

**Figure 3:**
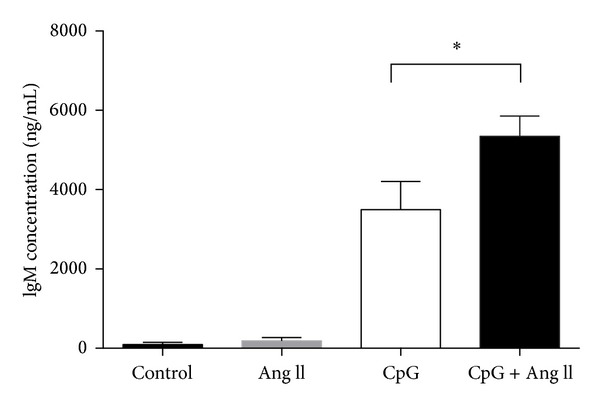
Effect of angiotensin II (Ang II; 0.1 *μ*M, 48 h) or CpG oligodeoxynucleotides (CpG; 5 *μ*g/mL, 48 h) alone or in combination on IgM antibody secretion from primary cultures of mouse B cells. Values represent mean ± S.E.M. of *n* = 14 experiments. *P < 0.05 for Bonferroni's post-hoc test after one-way ANOVA.

Hypertensive stimuli such as Ang II might act to directly modify the function of B cells, such that their capacity to produce antibodies is enhanced. Na^+^/H^+^ ion exchangers (NHEs) are critical regulators of intracellular pH and are crucial to a variety of fundamental cellular processes such as proliferation, growth, and migration [80]. Studies from the 1990s demonstrated that B cells isolated from a subset of hypertensive patients display heightened activity of NHEs [81, 82]. Moreover, these B cells were further characterised as having enhanced G-protein activation, a higher proliferative capacity, and augmented IgG and IgM antibody secretion compared to B cells from nonhypertensive individuals [83, 84]. While the mechanism underlying this increase in NHE activity in B cells was not explored, in other cell types (e.g., VSMCs and cardiomyocytes) it is known that AT_1_R stimulation can enhance NHE activity [85–87]. Indeed, B cells express AT_1_R [88] and we have preliminary data showing that stimulation of B cells isolated from mice with Ang II potentiates IgM formation in response to a known B cell stimulator, the oligodeoxynucleotide CpG (Figure 3). Thus, amplified antibody production may arise as a result of elevated NHE function due to the direct activation of AT_1_R expressed on B cells.

## 5. The Role of B Cells in Hypertension

Implicit in the previous discussion on antibodies in hypertension is a potentially important role for the cell type that produces antibodies, namely, B cells. In their seminal study, Guzik et al. showed that recombinase-activating gene-1 knockout (RAG1^−/−^) mice—which lack T and B cells—displayed a blunted hypertensive response to both Ang II and deoxycorticosterone acetate/salt [89]. Whereas adoptive transfer of T cells into RAG1^−/−^ mice recapitulated the full hypertensive effects of Ang II, transfer of B cells had no effect [89]. There are at least two potential explanations for the lack of effect of B cell adoptive transfer in Ang II-treated RAG1^−/−^ mice. First, it is possible that the adoptively transferred B cells did not engraft in sufficient numbers to influence immune function. Indeed, in a previous study it was shown that retroviral-mediated reintroduction of the RAG1 gene into RAG1^−/−^ mice restored T cells numbers back to levels in wild-type mice, while B cell numbers only increased marginally [90]. This suggests that the immunological environment in RAG1^−/−^ mice, while being favourable to the survival and function of T cells, may be incompatible with the growth and function of B cells. An alternative explanation may lie in the different mechanisms that activate T cells and B cells during an immune response. Whilst activation of T cells relies primarily on antigen presentation from innate immune cells such as dendritic cells (which are relatively unaffected in RAG1^−/−^ mice), as discussed previously, B cell activation and differentiation into an effector phenotype normally requires interactions with T_H_ cells [6]. Hence, the lack of T cells in RAG1^−/−^ mice may have precluded the possibility of any adoptively transferred B cells becoming activated. Indeed, a critical role of  T_H_ cells in B cell activation during hypertension was suggested in a recent study showing that adoptive transfer of T_H_ cells from preeclamptic mice into normal pregnant mice induced AT_1_-AA production and elevated blood pressure [91]. Importantly, a B cell depleting agent ameliorated both of these effects [91].

## 6. Therapeutic Implications

An understanding of the role of B cells and antibody production during hypertension could aid in the refinement of current treatment approaches and also in development of novel antihypertensive therapies. For example, by identifying the autoantibodies that are specifically elevated in a given hypertensive patient, it might be possible to “tailor” the way their disease is subsequently managed for better clinical outcomes; that is, individuals with AT_1_-AAs would favourably respond to AT_1_R blockers over patients with L-type VOCC autoantibodies, where calcium channel blockers such as nifedipine would be preferred.

In terms of new therapeutic approaches, identification of the specific pathogen-derived or neoantigens that lead to elevated antibody generation in hypertension could lead to immunisation strategies aimed at neutralising such antigens or steering the immune response away from one that promotes hypertension. Indeed, two vaccines against Ang II have been developed and showed some early promise in reducing blood pressure in hypertensive patients [92]. However, due to their lower efficacy compared to conventional inhibitors of the renin-angiotensin system, the vaccines did not proceed into Phase III clinical trials [93, 94], and thus further work is needed to determine if alternative immunisation strategies (i.e., involving different adjuvants and/or immunogens) will be more effective.

B cell-depleting agents, which include antibodies against the B cell specific surface receptor CD20 and the B cell activating factor BAFF, are already in clinical use for the treatment of autoimmune diseases such as lupus erythematosus [95] and could potentially be used to treat hypertension. Of course, these drugs have the potential for causing immunosuppression and hence their use might be best reserved for those hypertensive patients that do not respond to conventional therapies. Until recently, one of the main therapeutic options for individuals with resistant hypertension was surgical denervation of the renal artery [96]; however, the effectiveness of this procedure has recently been called into question [97]. Thus, B cell-modulating drugs might yet be a safer and more efficacious therapeutic option for such patients.

## 7. Conclusion

There is evidence that circulating antibody levels are elevated in both essential and pregnancy-related hypertension. Many of these antibodies appear to target receptors and ion channels known to be involved in the regulation of blood pressure. Further studies are required to characterise the precise impact that antibody binding has on the function of these proteins and to uncover the mechanisms responsible for aberrant antibody production in hypertension. Such studies should not only allow us to evaluate the significance of elevated antibody production in the pathophysiology of hypertension, but they may also lead to the development of new therapeutic approaches and/or the refinement of current approaches, to improve the management of clinical hypertension in the future.
